# Explaining the burden of psychosocial factors on the worsening symptoms of MS: a qualitative study of patients' experiences

**DOI:** 10.1186/s12883-023-03148-z

**Published:** 2023-03-06

**Authors:** Fahimeh Pourhaji, Nooshin Peyman, Mousa Mahdizadeh Taraghdar, Jamshid Jamali, Hadi Tehrani

**Affiliations:** 1 Department of Health Education and Health Promotion, Student Research Committee, Mashhad University of Medical Sciences, Mashhad, Iran; 2 Department of Health Education and Health Promotion, School of Health, Mashhad University of Medical Sciences, Mashhad, Iran; 3 Social Determinants of Health Research Center, Mashhad University of Medical Sciences, Mashhad, Iran; 4 Department of Medical Surgical Nursing, School of Nursing and Midwifery, Mashhad University of Medical Sciences, Mashhad, Iran; 5 Department of Biostatistics, School of Health, Mashhad University of Medical Sciences, Mashhad, Iran

**Keywords:** Multiple sclerosis, Psychosocial factors, Qualitative study

## Abstract

**Background:**

This study was conducted with the aim of identifying the burden of psychosocial factors on the worsening symptoms of multiple sclerosis.

**Methods:**

This as conducted with a qualitative approach and conventional content analysis among patients with Multiple sclerosis in Mashhad. Data were collected through semi-structured interviews with patients with Multiple sclerosis. Twenty-one patients with Multiple sclerosis were selected through purposive sampling and snowball sampling. The data were analyzed using Graneheim and Lundman method. Guba and Lincoln's criteria were used for evaluating research transferability. The data collection and management was performed by using the MAXQADA 10 software.

**Results:**

In explanation of the psychosocial factors of patients with Multiple sclerosis, one category (psychosocial tensions) and three subcategories of stress (physical symptoms, emotional symptoms, and behavioral symptoms), agitation (family disorder, treatment-related concerns, and social relationship concerns), and stigmatization (social stigma and internalized stigma) were extracted.

**Conclusion:**

The results of this study show that patients with Multiple sclerosis are faced with concerns such as stress, agitation, and fear of stigma, and need support and understanding from the family and community to overcome these concerns. Society must base its health policies on addressing the challenges faced by patients.

Accordingly, the authors argue that health policies, and consequently, healthcare systems, need to address patients’ ongoing challenges as a priority in caring for patients with Multiple sclerosis.

## Background

Multiple sclerosis (MS) is a chronic inflammatory autoimmune disease of the central nervous system (CNS). The disease leads to the formation of inflammatory lesions in the CNS in which myelin sheaths are broken and demyelinated axons are damaged. This leads to neurological symptoms such as impairment of motor function, sensitivity, balance, vision as well as fatigue, depression and cognitive changes [1]. Recent estimates show that 2.8 million people worldwide are living with this disease [2]. MS is usually diagnosed between the ages of 20 and 40 and is more common in women than men [3]. What is remarkable about MS in both developed and developing countries is the increase in its prevalence in recent years [4].

Today, despite significant advances in medical knowledge, the exact cause and method of treatment of this disease are still unknown. It has not been specified yet when the progression of the disease will slow down. In 85 to 90% of these patients, there are periods of recovery and exacerbation of symptoms [5].

The results of several studies show that at least one-third of patients experience a significant reduction in living standards after diagnosis. Within ten years of diagnosis, half of all patients with MS are unable to perform their daily tasks and obligations. Fifteen years after the disease onset, half of the patients need assistance to move [6].

A study showed that MS poses several challenges to physical and psychosocial health. MS can have profound consequences, including disruption of life goals, employment, income, relationships, social and leisure activities, and activities of daily living [7].

MS patients may experience social processes such as rudeness, indifference, humiliation, and rejection, these factors make the patient feel worthless, and life seems meaningless to them [8].

The lack of purpose and meaning in life leads to social withdrawal and isolation in MS patients [9].

Another study showed that patients with progressive types of MS had poorer quality of life, and were more likely to have marital problems, separation/divorce due to MS, and unemployment. Compared to others, they were five times more restricted while traveling in public places and experienced three times more constraints in social activities [10].

Increased likelihood of losing a job, reduced working hours or changing the type of work activity [10], loss of self-esteem, disruption of social interactions, feeling of disappointing their partner, social isolation, dependence on others in daily life, feeling powerless and inferior to others and functional limitations are factors that can threaten the dignity of people with MS as well as their life plan and goals [11]. Therefore; people with this disease need to adapt to its chronic challenges [12]. Due to the extensive socio-economic developments that have taken place in Iran in recent years, MS patients face many challenges in life due to the nature of their disease.

## Study aims

There are few studies conducted in Iran about the causes of these concerns, and these studies were all quantitative. Quantitative methods cannot always be used to understand meanings, beliefs, and experiences. Therefore; it is necessary to conduct a qualitative study for a deep understanding of the experiences, which provides a broad and rich description of the phenomena [13, 14]

Recently, studies of health issues have paid much attention to the qualitative approach. The main focus of qualitative approaches is interviewing participants. Qualitative studies provide appropriate insight into the semantic structure [15–17]. In addition, conducting this research in the Iranian culture can help clarify the burden of the psychological and social factors associated with the worsening symptoms of multiple sclerosis. Due to the complexity of MS, this qualitative study was conducted with the aim of identifying the burden of psychosocial factors on the worsening symptoms of multiple sclerosis.

## Methods

This study was conducted with a qualitative approach and conventional content analysis among.patients with MS in Mashhad, Iran (a large city located in the east of Iran), in 2022.

### Participants and recruitment

In this study, Sampling was selected purposefully, and until the data reached saturation, the sample selection process continued. In order to reflect diversity in a community, purposive sampling was used instead of statistical generalizability.

Participants were selected based on analysis of data, with maximum diversity in social status at the city level (from different geographical areas, urban and rural, of both sexes and different ages) were selected by referring to the MS comprehensive center and the MS association. The most important underlying factors of this design were sex, age, marriage, education level, occupation, and duration of illness. It should be noted that in the continuation of the research, the snowball sampling method was used to enter the research samples into the study. First, the necessary permits for the presence of the researcher at the comprehensive center and the MS Association were obtained. In the next step, the necessary arrangements were made with the officials of each department. The researcher then introduced himself, the objectives of the research, the importance of the research, and the conditions for entering the study while communicating with patients.

Finally, patients who wanted to be interviewed were interviewed after the necessary arrangements.

### Inclusion and exclusion criteria

The inclusion criteria consisted of Iranian patients with MS (according to the 2017 McDonald criteria [18]) who lived in Mashhad, it has been at least a year since they had been diagnosed with MS (by a physician), they signed the consent form entirely voluntarily and were interested in participating in the study. The exclusion criterion in this study was the unwillingness to continue cooperation.

### Ethical considerations

Prior to the interview, the objectives of the study were explained to the participants. Patients were assured that their information would remain confidential and would be used only for research purposes. Participants were informed that their voices needed to be recorded for the purpose of the study, and they were assured that their voices would remain confidential. After obtaining permission, participants’ voices were recorded. It is necessary to mention that this study was conducted following the Helsinki Declaration.

### Data collection and analysis

In this study, data were collected from patients with MS by using semi-structured interviews and face-to-face methods. After 18 interviews with patients, the data reached saturation because the codes were repeated, and new concepts were not extracted from the interviews. Three more interviews were conducted to ensure data saturation.

In this study, questions were asked, such as what psychological factors have contributed in aggravating your illness? Moreover what social factors have contributed to the aggravation of your illness? Has this disease affected your relationships with others? If yes, how?

At the end of the interview, participants were asked to talk about other matters that were not mentioned. Interviews were conducted in the MS comprehensive center affiliated with Mashhad University of Medical Sciences in conditions that the interview space was ideal in terms of ventilation, light, and sound.

The duration of each interview varies from 30 to 63 min, depending on the environmental factors and the tolerance level of the participant. The interviews were conducted between July and September 2022. During the interview, the researcher carefully observed all the participants' behaviors and, if necessary, took notes on their feelings, emotions, and reactions and, then, added these notes to the margins of the interview text when rewriting the interviews. Also, the contact information was provided to the participants so they could contact the researcher at another time if they wished to provide more detailed information.

### Methodological considerations

In this study, four standards of Lincoln and Guba (Validity, verifiability, portability, and reliability) were used to strengthen the data [19, 20]. In this study, the researcher first wrote down his beliefs and values, which she thought might influence the process of data collection and analysis, on paper in order to avoid applying her personal opinion and thoughts, and during the research tried to avoid emphasizing them. In this study, it was tried to increase the validity of the data by present in the research environment and continuous observation, interviewing a diverse range of patients and sharing the extracted codes and texts with several interviewees, and holding meetings with the supervisor team in order to review the typed materials resulting from the discussion with the participants. For the data to be verifiable, a part of the interpretations of participants’ explanations, by the participants themselves, and a part of the free codes and subsets by the experts have been checked, and the necessary corrections have been made. Data transferability was evaluated through purposive sampling and sampling with maximum diversity as well as data-saturated interviews. To increase data transferability, the researchers provided a detailed and step-by-step description of how to conduct the research. Additionally, the researchers presented the characteristics of the studied community to provide the possibility of following the process for other researchers. To the check reliability, the revision process was carried out in two stages by the research team: 1) Partial reliability control: revision of the category and coding guidelines after working with 10–50% of the data. 2) General reliability control: at the end of the work in order to catalog the final category.

Data analysis was performed using the Graneheim and Lundman approach. It consists of five steps: 1) writing down the entire interview immediately after conducting each interview, 2) reading the entire text several times to gain a general understanding of its content, 3) determining semantic units and basic codes, 4) classifying primary codes into more comprehensive categories, and 5) determining the main subject of categories [21]. First, after ensuring the participant's readiness and permission to record the interview, the interview was recorded with a mobile phone. Two recorders were used to ensure data back-up. After the interview, it was immediately transcribed verbatim on paper. Then the transcribed text was read several times. In the next step, the manuscript transcripts were typed and stored as the primary research data in Word 2016. The typed interview was entered into MAXQDA version 10 software and the data was analyzed with this software. Words and sentences (semantic units) were coded by a researcher, and open codes were formed. After extracting the initial codes, for integration, the items that were conceptually and semantically similar or were related to each other were placed in a category and formed a subcategory of a single topic. In this way, more comprehensive categories were formed and this process of analysis continued until the creation of the main and sub-categories.

**Fig. 1:**
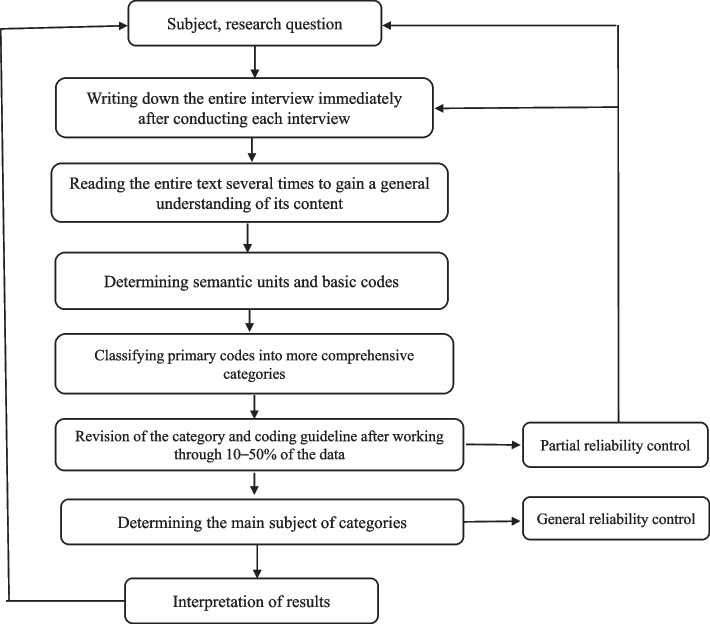
Flowchart of methodological steps

In order to review the codes, the help of an independent researcher was taken, and if there was an unresolved dispute between the first researcher and the independent researcher, the third researcher was involved in the discussion to make the final decision (Fig. 1).

## Results

This study was performed through semi-structured interviews with 21patients with MS. The mean and standard deviation of participants' age was 36.52 (± 7.06). The mean and standard deviation of the duration of the disease was 8.95 (± 6.25). Fifteen (71.4%) participants were female, and 6 (28.6%) were male. Three (14.3%) participants were single, 14 (66.7%) were married, and 4 (19.0%) were separated. One (4.8%) participant had an incomplete diploma, 8 (38.1%) had diplomas, one (4.8%) had an associate degree, 8 (38.1%) had bachelor's degrees, 1 (4.8%) participant had a master's degree, and 2 (9.5%) had doctorate.

In response to the question "What are the psychological and social concerns of people with MS?" one category (psychosocial tensions) and three subcategories of stress (physical symptoms, emotional symptoms and behavioral symptoms), agitation (family disorder, treatment-related concerns, and social relationship concerns) and stigmatization (social stigma and internalized stigma) were brought up. More complete results can be seen in Table 1.

**Table 1:** Categories and sub-categories extracted from the analysis of patients’ experiences

Main category	Sub-category	subcategory of subcategory	Free codes
**Psychosocial tensions**	Stress	Physical symptoms	Stiff hands, stiff legs, balance problems, crooked lips, blurred vision, central vision halos, double vision, uncontrollable tremors, inability to lift objects, Negative consequences of pregnancy,…
		Emotional symptoms	Feeling anger, feeling depressed, fatigue, reduced self-confidence, pessimism, the feeling of losing desires and dreams,…
		Behavioral symptoms	Slowing down of their daily activities, making mistakes in doing activities, sexual dysfunction, talk fast, …
	Agitation	Family disorder	Threat of divorce, marital disputes, changes in the dynamics of family members, the failure to meet the expectations of family members, …
		Treatment-related concerns	Drug shortages in pharmacies, drug embargoes, exorbitant drug costs, not having insurance, treatment related side effects, disappointment in the treatment, …
		Social relationship concerns	Concerns about impaired communication with the opposite sex, impaired communication with colleagues and superiors, losing prestige, fear of harming others at work, job threats, inability to communicate with others, the feeling of being misunderstood by others, transportation problems, …
	Stigmatization	Social stigma	Attributing inappropriate things and words to the patient, non-recognition of person's capability and competent to participate in the community, attributing the disease to other family members, fear of the patient, looking at the patient with a different perspective,.
		Self-stigmatization (Internalized stigma)	Feelings of helplessness, negative thoughts (being imperfect), being useless, annoying others, adding burden, being dependent on others,…

## Main category: psychosocial tensions

### First subcategory: stress

#### Subcategory of subcategory: physical symptoms

Participants reported that physical symptoms such as stiff hands, stiff legs, balance problems, crooked lips, blurred vision, central vision halos, double vision, uncontrollable tremors, inability to lift objects, and negative consequences of pregnancy were associated with worsening symptoms.

Participant No. 2 (Duration of illness 13 years): “One time, my lips were completely crooked. The doctor said that a muscle in one part of my face was involved, and my eye was also drooping. Every time an attack happens, it affects a different place, I am always afraid of these attacks. “

Participant No. 13 (Duration of illness 12 years): “… I had double vision and one side of my body was numb. I saw everything in doubles, meaning, I was walking on the street; I did not know which was the main car and which was the shadow car. The double vision was becoming very severe, oh God, what is happening? …”.

Participant No. 5 (Duration of illness 5 years): “… I was walking in the street, and for a moment, I felt that my left leg had become lightweight, I wanted to move, but my leg was not moving. Then I went home, I told myself that it probably got cold because it was snowing. I massaged it, warmed it up, then walked again and saw that, nope, I could not feel it.”

#### Subcategory of subcategory: emotional symptoms

Emotional symptoms such as anger, depression, fatigue, feelings of losing desires and dreams, pessimism, and reduced self-confidence were associated with worsening symptoms.

Participant No. 7 (Duration of illness 3 years): “… I feel like because we are sick, we think that if we say no once, every problem will end up being blamed on our illness”.

Participant No. 3 (Duration of illness 3 years): “… the others should increase their patience for a while, do not put pressure on us, know our situation, know that we will get tired fast, they should understand us, give us morale. Of course, their behavior should not be out of pity. They don't need to do anything special, they just to understand us.”

Participant No. 5 (Duration of illness 5 years): “… This is the only result I got from MS. The fact that someone with MS cannot control their nerves at all. At home, when my wife tells me something I immediately tend to lose my temper.”

Participant No. 16 (Duration of illness 13 years): “… I really wanted to continue my sport and reach high levels, but unfortunately, on the one hand, I didn't have the conditions to continue exercising, and on the other hand, supporting an MS patient is a difficult responsibility, and no one is willing to accept this responsibility.”

#### Subcategory of subcategory: behavioral symptoms

Participants reported various behavioral changes, such as Slowing down of their daily activities, making mistakes in doing activities, sexual dysfunction, and talk fast.

Participant No. 21 (Duration of illness 6 years): “… I get careless when I do a few things together at the same time. For example, I can still do those things, but it’s not the same as it was before my sickness. It affects my patience, and sometimes mistakes happen in my work that never happened before.”

Participant No. 2 (Duration of illness 13 years): “… I do not have the sexual desire at all. I studied a bit and found that MS itself affects sexual potency, as well as the drugs we use. However, this is a necessity of life, and I can’t say that there is none at all, but I find myself having little or no desire.”

Participant No. 20 (Duration of illness 20 years): “…When I talk, I seem anxious, I speak too fast and to be honest, I think it is not my fault, I have no control over my behavior, and unfortunately, everyone thinks that I am arguing with them.”

### Second subcategory: agitation

#### Subcategory of subcategory: family disorder

In this context, participants consider factors such as the threat of divorce, marital disputes, changes in the dynamics of family members, and the failure to meet the expectations of family members to be affective.

Participant No. 15 (Duration of illness 22 years): “… my husband remarried at the start of my illness, he cheated on me and many people told me to get a divorce, but I stayed because of my daughter.”

Participant No. 10 (Duration of illness 13 years): “… since I got sick, I can no longer go out and have fun with my children like before, the children became upset, they do not go out anymore."

Participant No. 1 (Duration of illness 15 years): “… My husband was always shouting, arguing, arguing with the children, grumbling, objecting to everything, I mean, he would not say directly what he was angry about, but he would lash out at everyone.”

#### Subcategory of subcategory: treatment-related concerns

Participants reported that drug shortages in pharmacies, drug embargoes, exorbitant drug costs, not having insurance, treatment related side effects, and disappointment in the treatment have contributed to the patients' agitation.

Participant No. 3 (Duration of illness 3 years): “… One of the problems we have is that when we take these medicines we have to go for a test later. This test is very expensive, and it really costs a lot of money for us. Sometimes when I go to the pharmacy, they say, they don’t have the medicine.”

Participant No. 20 (Duration of illness 20 years): “… At the moment, they haven’t found a cure yet. I hope that someday they fully understand this disease and there will be a cure for it. Everyone you talk to says that it is an autoimmune disease and everything is based on probability.”

#### Subcategory of subcategory: social relationship concerns

In this subcategory, concerns about impaired communication with the opposite sex, colleagues, superiors, fear of harming others at work, inability to communicate with others, the feeling of being misunderstood by others, job threats and transportation problems were associated with worsening symptoms.

Participant No. 4 (Duration of illness 5 years): “… I was in an emotional affair with the opposite sex, then the disease started right away, because most of my worries were about that emotional affair, I was always afraid that my boyfriend would find out. In the end, the boyfriend has a right to choose, that is, if they find out that their partner has this problem, they can very well set aside their partner and they have a right to choose.”

Participant No. 2 (Duration of illness 13 years): “… Most co-workers are not supportive, everyone is looking for their own interests and if find out that you are sick, they may use that fact to their advantage.”

Participant No. 11 (Duration of illness 5 years): “… No one can make me feel better, that is, neither better nor worst. I don't talk to anyone much; I don't confide in anyone. Actually, there is no one who is aware of this disease and this issue, that you are able to confide in. Everybody says: ‘Oh, did you know that this other guy has it too’, and they just talk behind your back and don’t help at all.”

Participant No. 15 (Duration of illness 22 years): “… what prevents you from going to classes is being too far away because it is difficult to walk, or for example, in the heat and cold, you can’t go out without a vehicle. Many times motorcycles and people become bothersome. Commuting has become downright difficult for me.”

### Third subcategory: stigmatization

#### Subcategory of subcategory: social stigma

In this regard, patients reported that attributing inappropriate things and words to the patient, non-recognition of person's capability and competent to participate in the community, attributing the disease to other family members, fear of the patient, looking at the patient with a different perspective adds to the aggravation of the disease.

Participant No. 18 (Duration of illness 6 years): “…. nobody proposes to my sisters, everyone thinks that if I’m sick, then the rest of my sisters will also get sick, and that their son will get into trouble and have a sickly wife that they have to pay for their medicines and treatments.”

Participant No. 14 (Duration of illness 9 years): “… People’s looks bother me, they look at me like they think that I have leprosy, because of this behavior sometimes I do not go to the doctor.”

Participant No. 20 (Duration of illness 20 years): “…. One time, one of my colleagues kept bothering me about why my hand is like this. He said, 'Wow, why is your hand shaking?' After that I tried to not be around him so that I do not cross paths with him”.

Participant No. 19 (Duration of illness 5 years): “I would like that when I cannot walk, they do not look at me like I’m a sick person or look at me with pity.”

#### Subcategory of subcategory: self-stigmatization (Internalized stigma)

Feelings of helplessness, negative thoughts (being imperfect), being useless, annoying others, adding burden, being dependent on others, were associated with worsening symptoms.

Participant No. 20 (Duration of illness 20 years): “….I don't think about marriage so that I don't let anyone into my life, because he (she) will also be bothered and burdened by my pain and problems, I do not want anyone to enter my life, they do not have the capacity to deal with my problems and hardships, they even blame a healthy person let alone a sick one. When we get married they will say you brought all these pain and sufferings with you”.

Participant No. 12 (Duration of illness 4 years): “…. whatever you do, you are still the same sick person, for example, they told me to occupy myself and go to classes, I was occupying myself and I was doing housework, but it was of no use. The person who gets sick will never be the same person they were before. I am sad about my fate and I cry a lot.”

## Discussion

The aim of this study was identifying the burden of psychosocial factors on the worsening symptoms of multiple sclerosis. Based on the results of this study, three subcategories of stress (physical symptoms, emotional symptoms and behavioral symptoms), agitation (family disorder, treatment-related concerns and social relationship concerns) and stigmatization (social stigma and internalized stigma) were addressed.

The results of this study show that stressors ranging from stress caused by physical disabilities to emotional and behavioral which is caused by dysfunction in normal daily activities are associated to the recurrence of MS. The patients reported that physical insufficiency caused by the disease, especially the fear of falling and balance problems are among the stressors that contribute to the exacerbation of the disease. In this regard, other studies show that MS patients are afraid of falling and walking disorders [22, 23].

The results of a study showed that physical disabilities have many complications in the daily lives of patients with MS. For example, the nature of the disease limits their activity at work, at home, and in the community, and therefore places a significant burden on people with MS [24].

In another study, the patients stated that they were afraid of pregnancy, and that the effects of pregnancy on MS were worrying for women with MS. They were also concerned about the short-term and long-term effects of pregnancy on MS, the effects of MS on pregnancy, the effects of MS treatments on pregnancy, and the possible mechanisms of these effects [25–27].

The results of this study showed that the occurrence of emotional symptoms were associated with worsening symptoms. The results of studies show that emotional stressors are related to the exacerbation of neurological symptoms [28]. Studies have shown that depression, fatigue, cognitive disorders, and sleep disorders are common symptoms that negatively affect daily life and social and professional activities [24, 29].

The results of Studies show that fatigue is the most common symptom reported by MS patients. The fatigue is a subjective feeling that brings a significant physical and psychological burden, especially during daily tasks [30].

The results of this study showed that patients with MS exhibited signs of behavioral changes. In this regard, a study showed that behavioral changes are common in patients with multiple sclerosis [31].

Jasminka Djelilovic-Vranic showed that stress due to disability in the workplace and interpersonal relationship problems are effective in exacerbating MS disease [32].

Rocco Salvatore Calabr J in their study showed that one of the most common concerns in MS patients is related to sexual dysfunction. Sexual dysfunction is a very common and devastating problem in people with MS, including young patients with mild disabilities [33]. The results of studies on the prevalence of perceived sexual anxiety in MS showed that 40 to 80% of women and 50 to 90% of men have sexual complaints [34].

The participants also stated that they could not do their routine tasks that they used to be able to do, which affected both their relationships with others and their self-esteem [35].

The results of Oveisgharan, Shahram’s study showed that stressors are associated with the severity of MS [36].

The loss of patients' sensory-motor abilities and the future of their functional capabilities is another source of stress that feeds patients' fear of relapse. Chronic illness is associated with patients' long-term stress and fear of things related to the sources of stress [37].

Therefore, the family and the community should play a supportive role for an MS patient and include stable fields of mental care such as counseling and psychology classes in the patient’s schedule, so that the mental state of the patient can be continually assessed and the best approach to "overcoming stress" be implemented in the patient.

## Agitation (Family disorder, treatment-related concerns and social relationship concerns)

The results of the Soodeh Maghsoodi’s study show that MS plays a major role in family conflicts, and the experience of rejection, abandonment, and divorce is one of the experiences that people with MS fear and stress over [38].

The results of a study showed that patients may not be able to participate in activities that they previously enjoyed for a long time, and this often changes the dynamism and mobility of the family [39].

The results of another study showed that family members may experience feelings of sadness with each new symptom and change in their patient's ability to function, many of which may affect family roles and change their future plans [40]. Also, reports indicate that MS leads to a decrease in people's abilities to fulfill social roles [41].

The results of another study showed that MS has a significant effect on families and affects their well-being and quality of life and often causes psychological stress in each member of the family and also in the overall performance of the family [42].

In case of the subcategory of treatment-related concerns, drug shortages in pharmacies, drug embargoes, exorbitant drug costs, drug efficacy, drug substitution, treatment related side effects, and disappointment in the treatment have contributed to the patients' agitation.

Mitra Habibi in her study showed that the access to effective drugs in treating MS is often limited due to their high cost and limited distribution [43].

The results of Mohammad Ali Sahraian's study showed that the majority of MS patients reported a simultaneous increase in care and treatment costs and concerns about reduced access to medications. The sanctions of drug affect the availability and costs of drugs, leading to a significant population of MS patients being forced to replace internationally branded drugs with national alternative drugs, while many of them have concerns about the effectiveness of national drugs [44].

The results of a study showed that drug therapy is effective in managing the pain caused by MS, but is often associated with several side effects that cause disruption in their daily lives such as arrhythmia, dizziness, imbalance, increased fatigue, etc. [45].

The results of another study showed that the available treatment options for progressive multiple sclerosis are relatively disappointing and still remain a challenge [46].

The results of the study showed that social relationship concerns contribute to the development of agitation in patients. In one study, participants indicated that MS was involved in ending a romantic relationship with the opposite sex, and also stated that a healthy person will be afraid of the possibility that being in a relationship with a person with MS will become a heavy burden [35].

The results of a study showed that the employment status is negatively affected by disability, fatigue and cognitive impairment of MS [47].

The results of the S. El-Wahsh’s study showed that the patients reported that MS led to changes in communication. Communication changes affect interpersonal relationships, participation, and identity in career paths and the workforce and higher education [48].

In another study, people with MS report that the workplace is often unsupportive. People with MS often hid the disease in their workplace and did not want their co-workers to know, as it may lead to a loss of co-worker trust and social isolation in the workplace [49].

The results of Leslie Beth Herbert's study showed that the feeling of not being understood by others is often seen as an obstacle for the participants' willingness to communicate with others. The participants reported feeling isolated and deliberately avoiding others because of MS [35].

The results of a study showed that at the beginning of the disease, patients experience fundamental life changes. They experience a reduction or loss of important competencies and abilities, which used to give them a sense of dignity. Violation of dignity means loss of self-esteem, pride and sense of worth which makes people feel bad about themselves [8–11].

The results of another study showed that many patients experienced a fear of harming others because of the type of work they did (For example: truck driver, electrician and child care provider) [50].

The results of studies show that losing a job has far-reaching financial, emotional, and social consequences, but is a common experience for people with MS. About half of patients who quit do so within three years of diagnosis. People with MS are concerned about their ability to manage their work life, and their work life becomes a source of anxiety and loss of confidence for them [51].

## Stigmatization (social stigma and internalized stigma)

The results of a study showed that most people with MS experience social stigma as a result of their MS at mild to moderate levels [52] Multiple sclerosis patients often feel rejected, judged and worthless in the eyes of others due to the disease [53].

Another study also showcased that patients with higher levels of disability have a higher perception of stigmatization. There is also evidence that stigma is an issue even in people with mild disabilities. As a result, stigma may be an important factor on the results of studies about MS [45].

The results of a study showed that from the moment people are diagnosed with the disease, they realize that they have a chronic disease that in some ways affects the way others see and targets them potentially [54].

The daily symptoms and disabilities that people with MS experience may lead to embarrassing public situations and consequent social isolation. For example, some people with MS rely on assistive devices such as crutches or wheelchairs for mobility. Such aids are associated with social stigma and presumed disability [24].

The results of Maria Anagnostouli showed that the state of disability affects the internalized stigma, the disability facilitates the thoughts of worthlessness and guilt for the development of MS in the affected person [55].

Feeling overwhelmed, especially financially, in people who do not have an independent income becomes a problematic factor, resulting in a significant reduction in social dignity of MS patients [38].The results of a study showed that decreased self-esteem and self-efficacy are consequences of internalized stigma [52].

In order to change the misconceptions among family members, others and the general public, proper solutions need to be implemented so that MS patients can achieve social approval and get rid of the stigma caused by this disease.

Therefore, it is necessary to further investigate these misconceptions and correct them through mass education via using the mass media. Policies should be designed to provide appropriate educational interventions to change certain behaviors. Factors that threaten the dignity of MS patients should be identified and appropriate measures should be taken to moderate these factors.

### Clinical implications

Therefore, it suggested that a multi-level intervention program designed in the first step; so that individual-level intervention programs can reduce stress and internal stigmatization in patients through education. Further, intervention programs at the family level can improve acceptance of the disease, and how to face and help the MS patient through education.

The level of community programs can provide necessary education to lead regular social communications, inappropriate beliefs, and social stigma caused by disease. It seems that intervention programs in the level of structure can play a crucial role in changing health policies to increase access to medicines and disease-modifying therapies.

## Research limitations

The results of this study are limited to the community of MS patients in the Iranian culture.

Therefore, to benefit from the findings of this study, it is necessary to conduct further studies in different cultures and fields. Despite the researcher's efforts to observe the principle of neutrality in the interview session, sometimes, this principle may not have been observed inadvertently.

Further research is needed to provide more complete insights into the psychological and social factors associated with MS. It is possible that the improvement of self-care helps to reduce the psychological and social challenges of patients; therefore, research to investigate the effect of self-care training on reducing psychosocial challenges can be useful.

## Conclusion

The results of this study showed that psychosocial factors experienced by patients play a role on the worsening of MS symptoms.

MS patients are faced with concerns such as stress, agitation and fear of stigma, the patient needs support and understanding from family and community to overcome these challenges. The family and community must have the ability to improve their level of understanding, update their information, and have the necessary knowledge and skills to both deal with and help the patients.

They also need a society whose health policies are based on addressing the challenges faced by patients and policies that provide appropriate care and clinical interventions, develop counseling programs for the patient and the patient's family, use media to share information, guarantee access to equal information for disease modifying therapies, and highlight the financial and social burdens of the MS disease.

Accordingly, the authors argue that health policies, and consequently health care systems, need to address the challenges that were mentioned in this study as a priority in caring for MS patients.

## Data Availability

The data sets used and/or analyzed during the current study was available from the corresponding author on reasonable request.

## Abbreviations

MS: Multiple sclerosis
CNS: Central Nervous System
